# Establishment of a High-Frequency Plant Regeneration Protocol for the Multipurpose *Handroanthus chrysanthus*

**DOI:** 10.3390/plants15132078

**Published:** 2026-07-03

**Authors:** Huiting Fang, Bin Chen, Junjie Zhang, Jiwen Zha, Xinwen Xu, Yutong Liu, Changcao Peng, Xiaolan Zhao

**Affiliations:** 1 Guangdong Key Laboratory for Innovative Development and Utilization of Forest Plant Germplasm, South China Agricultural University, Guangzhou 510642, China; 2 Zhongshan Innovation Center of South China Agricultural University, Zhongshan 528400, China; 3 Heishiding Nature Reserve of Guangdong Province, Zhaoqing 526536, China

**Keywords:** *Handroanthus chrysanthus*, direct organogenesis, explant type, growth regulators, micropropagation

## Abstract

*Handroanthus chrysanthus* (Jacq.) S.O. Grose is a Neotropical tree species highly valued for its ornamental beauty, durable timber, and medicinal properties. However, overexploitation and the recalcitrant nature of its seeds have constrained propagation and conservation efforts, and the species has been listed as Vulnerable on the IUCN Red List since 2020. In this study, a direct adventitious shoot regeneration system was developed for *H. chrysanthus* by systematically evaluating explant type, basal medium, plant growth regulator combination, and light quality. Hypocotyls were identified as the most responsive explants for shoot induction, and the highest adventitious shoot induction frequency, 51.79%, was obtained on Murashige and Skoog medium (MS) supplemented with 5 mg·L^−1^ 6-benzylaminopurine, 0.5 mg·L^−1^ indole-3-butyric acid, and 0.2 mg·L^−1^ thidiazuron under white fluorescent light. The highest shoot multiplication coefficient, 3.27, was obtained on MS medium containing 4 mg·L^−1^ 6-benzylaminopurine and 0.3 mg·L^−1^ gibberellic acid. The maximum rooting frequency, 80%, was obtained on R14 medium after 30 days of culture. After acclimatization, 95% of the regenerated plantlets survived and grew vigorously under greenhouse conditions. To our knowledge, no direct adventitious shoot regeneration system has been reported for the Tabebuia alliance. This efficient regeneration protocol provides a practical platform for clonal propagation, germplasm conservation, and genetic improvement of *H. chrysanthus* and may also support regeneration and conservation in related endangered taxa within the Tabebuia alliance.

## 1. Introduction

*Handroanthus chrysanthus* (Jacq.) S.O. Grose (syn. *Tabebuia chrysantha*), commonly known as golden-bell wood, is a deciduous tree belonging to the *Tabebuia* alliance (Bignoniaceae). The alliance currently includes approximately 99 species across three genera, although these taxonomic numbers remain provisional because the classification of the Tabebuia alliance is still under revision based on ongoing molecular phylogenetic studies [1,2,3]. Many species, including *H. chrysanthus*, are renowned for their exceptionally durable timber (Ipê) and spectacular floral displays [4,5,6]. However, overexploitation has led to indiscriminate logging [7], and the species has been listed as Vulnerable on the IUCN Red List since 2020 [8].

*H. chrysanthus* was introduced into the subtropical regions of southern China over 40 years ago [5,6]. Currently, *H. chrysanthus* is primarily propagated by seeds. However, this approach encounters two major constraints. First, fruit maturation coincides with the rainy season in southern China, and the winged seeds are prone to dispersal and mold. Second, as a recalcitrant species, its seed viability declines rapidly after harvest—germination rates remain viable for approximately 60 days and drop to nearly zero after three months [9]. These limitations hinder large-scale propagation and ex situ conservation.

Plant tissue-culture technology offers many advantages—such as high reproduction efficiency, stable seedling growth, and seasonally unrestricted propagation—to effectively meet market demand. Moreover, in vitro micropropagation serves as a foundational technology in both conventional and modern breeding, enabling polyploid development, elite trait selection, and genetic transformation of recalcitrant tissues [10], thereby significantly accelerating the breeding process to improve undesirable traits such as short flowering period and cold sensitivity.

*H. chrysanthus* has also been used in traditional medicine for treating fever, inflammation, and pain [11,12,13,14]. Pharmacological studies have confirmed its anti-inflammatory, antimicrobial, and anticancer activities [15,16,17,18,19,20]. However, these medicinal properties are not the focus of the present study. In the *Tabebuia* alliance, the micropropagation protocols of *H. heptaphyllus* [21], *H. impetiginosus* [22,23,24,25], *T. donnell-smithii* [26], and *H. guayacan* [27] have been previously established using nodal segments from in vitro-germinated seedlings or adult plants. More recently, Héctor [7] developed a micropropagation protocol for *H. chrysanthus* using apical buds from seed-derived plants and achieved a maximum propagation coefficient of 3.35 on WPM (Woody Plant Medium) medium supplemented with 6.6 μM 6-BA.

However, to date, there have been no reports on the establishment of a direct adventitious shoot regeneration system within the Tabebuia alliance. Unlike nodal micropropagation, which is limited to clonal propagation, an adventitious shoot regeneration system provides a basis for genetic transformation (Agrobacterium-mediated transformation or CRISPR/Cas9), enabling targeted trait improvement such as flowering regulation or cold tolerance [10]. In this context, the main aim of this research was to study the direct organogenesis potential of *H. chrysanthus*. The specific objectives of this study were to investigate the effects of key factors on direct shoot regeneration in *H. chrysanthus*, including explant type (cotyledons and hypocotyls) and medium composition (basal culture media and plant growth regulators (PGRs)). This established regeneration protocol will facilitate large-scale propagation of *H. chrysanthus* and serve as an essential technical foundation for genetic improvement and accelerated breeding of new cultivars of *H. chrysanthus*. The optimized regeneration system described here also provides a technical reference for related species within the Tabebuia alliance, an economically significant lineage of Neotropical trees.

## 2. Results

### 2.1. Initial Aseptic Culture Establishment

In this study, the seeds of *H. chrysanthus* from six disinfection treatments were cultured on hormone-free MS basal medium. After 20 days, the contamination and germination rates recorded after 20 days are presented in Table 1. Among the six treatments evaluated, surface sterilization with 75% ethanol for 50 s followed by 0.1% HgCl_2_ for 10 min was the most effective for *H. chrysanthus* seeds, yielding a high germination rate of 86.32% with only 10.20% contamination. Longer exposure (16 min) reduced contamination but significantly decreased germination, consistent with the findings of Zha et al. [25], who observed similar HgCl_2_ toxicity in *H. impetiginosus*. Although a small number of seeds did not germinate, most disinfected seeds of *H. chrysanthus* germinated on hormone-free MS basal medium and developed into normal seedlings (Table 1). This finding contrasts with the previous report by Héctor [7], who suggested that MS medium was not suitable for the germination of *H. chrysanthus* seeds. The discrepancy may be related to differences in seed provenance. In Héctor’s study, seeds were collected from natural populations in Ecuador, whereas those used in the present study were obtained from cultivated trees in Guangzhou, China. These cultivated trees originated from populations introduced into subtropical China several decades ago [5,6], and long-term cultivation under local environmental conditions may have altered their seed germination characteristics and response to MS medium.

### 2.2. Effect of Plant Growth Regulators on Shoot Regeneration from the Hypocotyls of H. chrysanthus

#### 2.2.1. Effect of 6-BA and NAA on Shoot Regeneration

**Figure 1:**
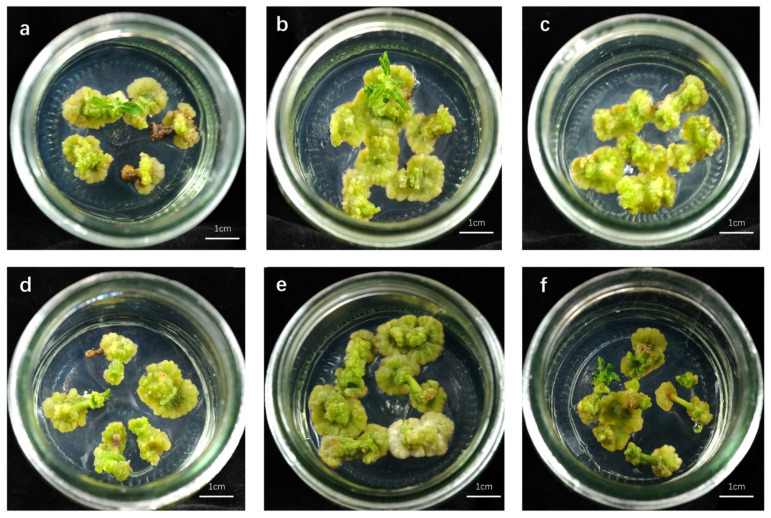
In vitro culture of *Handroanthus chrysanthus* hypocotyls under different combinations of 6-BA and NAA after 35 days: (**a**–**f**) Callus and shoot regeneration from hypocotyls in treatments SR2, SR5, SR8, SR9, SR10, and SR11, respectively. Scale bar 1 cm.

As shown in Table 2 and Figure 1, adventitious shoots were induced from hypocotyl explants cultured on MS basal medium supplemented with different concentrations of 6-BA and NAA (1-naphthaleneacetic acid). After seven days of culture, both ends of the hypocotyls began to swell, followed by the formation of compact green callus. The callus morphology, including growth rate, texture, and color, as well as the efficiency of shoot regeneration, varied significantly depending on the combination of 6-BA and NAA (Figure 1).

Adventitious shoot formation was first observed at 20 days of culture in SR6 medium (MS + 5.0 mg·L^−1^ 6-BA + 0.1 mg·L^−1^ NAA), with shoots emerging from one end of the hypocotyl explants. In SR5, SR10, and SR12 media, shoots appeared around day 25, whereas in the remaining formulations, the induction took 35 to 45 days. After 45 days, MS medium containing 5.0 mg·L^−1^ 6-BA with 0.05–0.1 mg·L^−1^ NAA (SR5 and SR6) produced adventitious shoots at a frequency of approximately 20% (Table 2).

#### 2.2.2. Effect of TDZ, 6-BA, and IBA Combinations on Shoot Regeneration

**Figure 2:**
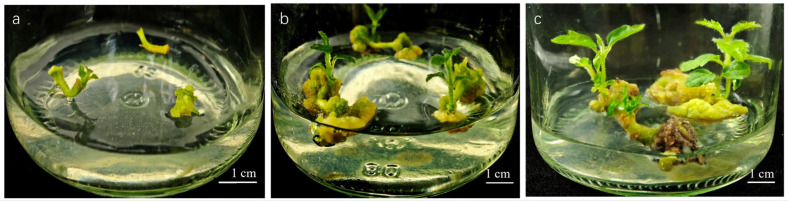
Adventitious bud regeneration process from *Handroanthus chrysanthus* hypocotyls in SI13 media: (**a**–**c**) The SI13 culture media contained 5 mg⋅L^−1^ 6-BA + 0.2 mg⋅L^−1^ TDZ + 0.5 mg⋅L^−1^ IBA. Hypocotyls cultured in Medium SI13 for 20 d, 45 d, and 60 d, respectively. Scale bar 1 cm.

Thidiazuron (TDZ) is a phenylurea-derived compound widely recognized as a highly effective cytokinin for inducing adventitious shoot regeneration in recalcitrant woody plant species [28,29]. To improve the regeneration efficiency of *H. chrysanthus*, we evaluated the effect of TDZ, 6-BA, and IBA (indole-3-butyric acid) combinations on adventitious shoot regeneration. Initial morphological changes were observed within 5 days of culture, with explants showing swelling at both ends or throughout their length, followed by callus formation at the cut. Shoot initiation first occurred in medium SI3 at 15 days, followed by media SI10, SI12, and SI13 at 20 days, while the remaining media showed shoot formation between 30 and 40 days. Among these, medium SI13 (MS supplemented with 0.2 mg·L^−1^ TDZ, 5.0 mg·L^−1^ 6-BA, and 0.5 mg·L^−1^ IBA) achieved the highest regeneration frequency of 51.79% (Table 3). Figure 2 illustrates the adventitious bud regeneration process from *H. chrysanthus* hypocotyls in SI13 media. Notably, at constant IBA (0.5 mg·L^−1^) and TDZ (0.2 mg·L^−1^) concentrations, increasing 6-BA levels enhanced both shoot regeneration and multiplication. However, when 6-BA (1.5 mg·L^−1^) and IBA (0.5 mg·L^−1^) concentrations were maintained, elevated TDZ levels progressively reduced shoot regeneration rates, indicating that excessive TDZ concentrations negatively impact regeneration efficiency in *H. chrysanthus* hypocotyl cultures.

### 2.3. Effect of Light Spectra on Shoot Regeneration from the Hypocotyls of H. chrysanthus

To investigate the effects of light spectra on the adventitious bud regeneration rate of hypocotyls, *H. chrysanthus* hypocotyl explants were cultured horizontally on MS medium supplemented with 5 mg·L^−1^ 6-BA, 0.2 mg·L^−1^ TDZ, and 0.5 mg·L^−1^ IBA under white, red, green, or blue light-emitting diode (LED) light. White LED light proved most effective, yielding the highest shoot induction rate (51.79%) and the greatest number of shoots per explant (2.20). In contrast, green light completely inhibited shoot organogenesis, with no adventitious shoots observed throughout the culture period (Table 4).

### 2.4. Effects of Basal Media and Plant Growth Regulators on Shoot Regeneration from the Cotyledons of H. chrysanthus

**Figure 3:**
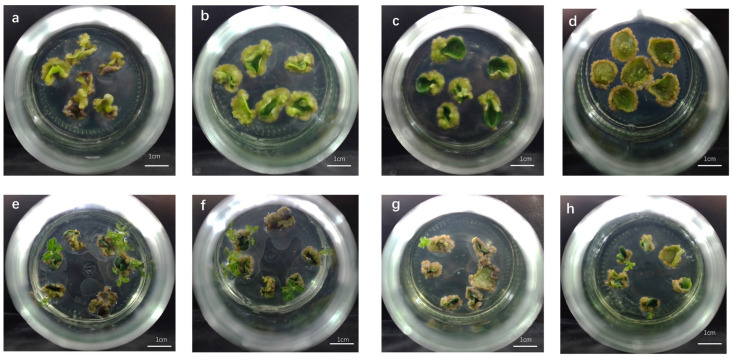
Adventitious bud induction and growth of *Handroanthus chrysanthus* cotyledons in different culture media. Scale bar = 1 cm: (**a**) DC1; (**b**) DC3; (**c**) DC5; (**d**) DC7; (**e**) DC2; (**f**) DC4; (**g**) DC8; (**h**) DC9.

In preliminary experiments, cotyledon explants of *H. chrysanthus* were excised and cultured with their abaxial side in contact with either MS [30] or Douglas-fir Cotyledon Revised (DCR) [31] basal medium, each supplemented with various concentrations of 6-BA and NAA. However, adventitious shoot regeneration was observed only in cultures on DCR-based media; all MS-based treatments failed to induce adventitious shoot generation from cotyledon explants. Therefore, Table 5 only presents the results using DCR as the basal medium. With its lower inorganic salt content and relatively mild osmotic environment, DCR medium effectively supports adventitious bud formation in various woody plants through either indirect organogenesis or direct regeneration pathways [31,32,33]. After 60 days of culture, the frequencies of callus and adventitious bud induction, as well as the number of induced buds, were determined, and the results are shown in Figure 3 and Table 5.

After 9 days of culture, the cotyledon explants began to swell and formed the callus at the wounded cuts. Adventitious bud differentiation was first observed around day 35 in five media: DC2, DC4, DC7, DC8, and DC9. The highest bud induction rate of 25.00% was achieved in the DCR medium containing 5 mg·L^−1^ 6-BA and 0.05 mg·L^−1^ NAA (DC4). As illustrated in Figure 3e, explants cultured on this medium produced yellow-green callus at the cut, from which clusters of robust, green shoots with well-expanded leaves emerged. The relatively high variability in shoot induction from cotyledon explants (e.g., DC4: 25.00% ± 5.88) may be attributed to explant heterogeneity, including the differences in physiological state and endogenous hormone levels among individual cotyledons from different seeds. Interestingly, increasing the 6-BA concentration (as in medium DC7) resulted in a significantly lower shoot induction rate compared to DC4, indicating that higher cytokinin levels are not beneficial for cotyledon regeneration in *H. chrysanthus* (Table 5).

To our knowledge, this study represents the first successful report of in vitro adventitious shoot regeneration for the Tabebuia alliance. Our results demonstrated that *H. chrysanthus* hypocotyl-derived cultures consistently outperformed cotyledon explants, including induction frequency (51.79% versus 25.00%) and response time (15–20 days versus 30–40 days). Explant-dependent variation in regeneration potential is well documented among woody species, including Eucalyptus [34] and a range of other taxa [28]. Specifically, hypocotyl segments have consistently demonstrated greater regenerative capacity compared to cotyledonary explants.

### 2.5. Effect of 6-BA and GA_3_ on Shoot Proliferation

Following the establishment of an adventitious bud induction system for *H. chrysanthus* hypocotyls, we further investigated a rapid micropropagation protocol using the shoots. Ideally, the adventitious shoots obtained from the induction experiment (Section 2.2) should be used for proliferation optimization. However, the optimal induction treatment (SI13) yielded a regeneration rate of only 51.79%, and the number of available adventitious shoots was insufficient to support the multi-treatment, multi-replicate proliferation experiments. Therefore, as an alternative, we used seedling-derived nodal segments (with axillary buds) as explants for proliferation optimization, a strategy commonly employed in similar studies [7,21,22,23,24,25,26,27]. Although we have developed a micropropagation system for seedling-derived explants of *H. impetiginosus*, this protocol did not achieve satisfactory multiplication rates when applied to *H. chrysanthus*. Moreover, using the medium formulation (WPM supplemented with 6.6 μM 6-BA) reported by Héctor [7], we could only obtain a proliferation coefficient of 1.8. To optimize the rapid propagation protocol of *H. chrysanthus*, we employed MS basal medium supplemented with various combinations of 6-BA (2, 3, and 4 mg·L^−1^) and GA_3_ (0.1, 0.3, and 0.5 mg·L^−1^) based on the study by Cardoso and Silva [35] on the micropropagation of *Zeyheria montana* (Bignoniaceae).

**Figure 4:**
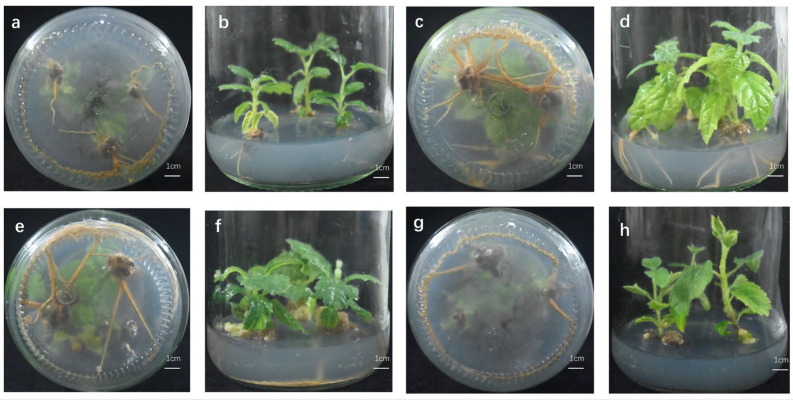
Effects of different hormone concentrations on rooting and growth of *Handroanthus chrysanthus* micropropagated seedlings. Representative shoot and root growth of *H. chrysanthus* micropropagated seedlings at 30 days in R1 (**a**,**b**), R11 (**c**,**d**), R14 (**e**,**f**), and R16 (**g**,**h**).

**Figure 5:**
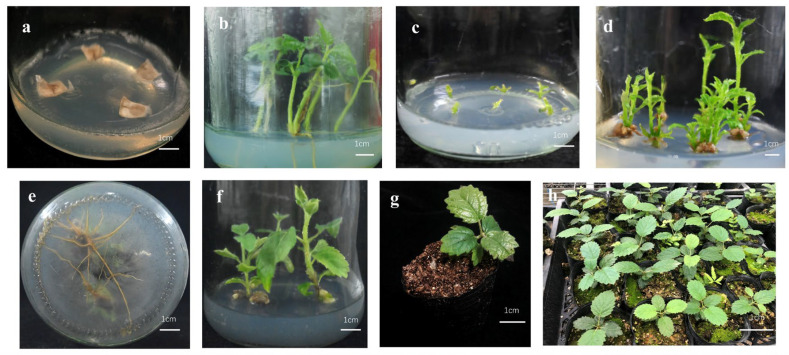
Micropropagation procedure of *Handroanthus chrysanthus*: (**a**) seed surface sterilization and inoculation in vitro; (**b**) regenerated shoots after 90 days of culture; (**c**) induction stage of stem segments with axillary buds on MS medium supplemented with 4 mg⋅L^−1^ 6-BA and 0.3 mg⋅L^−1^ GA_3_ (SP6); (**d**) shoot proliferation on SP6 medium after 45 days; (**e**,**f**) rooting of regenerated shoots after 30 days of culture; (**g**) acclimatization of rooted plantlets at 10 days after transplantation; (**h**) growth condition after transplantation for 60 days.

As shown in Table 6, all media formulations yielded a proliferation coefficient exceeding 2.1, indicating that the combination of 6-BA and GA_3_ is beneficial for the rapid propagation of *H. chrysanthus*. The optimal medium for shoot proliferation was MS basal medium containing 4 mg·L^−1^ 6-BA and 0.3 mg·L^−1^ GA_3_ (SP6), which resulted in a proliferation coefficient of 3.27 and an adventitious bud induction rate of 91.67%. The proliferated shoots grew vigorously with well-expanded leaves (Figure 4 and Figure 5).

### 2.6. Rooting and Acclimatization of Micropropagated Seedlings

Half-strength MS basal media supplemented with IBA and/or NAA were evaluated for their potential in inducing root formation (Table 7). When IBA was used alone, the rooting percentage was only approximately 30% after 30 days, whereas the combination of NAA and IBA significantly promoted the rooting process (Figure 4). The highest rooting rate of seedlings reached 80% after 30 days in medium R14 (1/2 MS + IBA 5 mg·L^−1^ + NAA 0.5 mg·L^−1^ + activated charcoal (A.C.) 0.2 g·L^−1^). Based on a comprehensive evaluation of rooting percentage and the average root number per plant, R11 (1/2 MS + IBA 10 mg·L^−1^ + NAA 0.05 mg·L^−1^ + A.C. 0.2 g·L^−1^) represented another preferable medium.

The vigorous plantlets with 2–3 cm root length were transferred to room temperature under natural light for 3–4 days. The planting substrate consisted of a sterilized mixture of peat soil, vermiculite, and perlite (3:1:1). During transplantation, plantlets were gently removed from containers, and the planting trays were covered with transparent lids and maintained in a greenhouse for 7 days before removing the covers. Under natural conditions, the survival rate reached 95% after one month. Figure 5 illustrates the successful micropropagation process of *H. chrysanthus*.

## 3. Discussion

In the present study, medium containing 6-BA combined with NAA (SR5 and SR6) yielded a regeneration frequency of approximately 20%, whereas the combination of TDZ, 6-BA, and IBA (SI13) significantly enhanced regeneration to 51.79% from *H. chrysanthus* hypocotyls. TDZ exhibits potent cytokinin-like activity while also modulating endogenous auxin metabolism, thereby stimulating the accumulation of natural cytokinins and promoting morphogenic responses [36,37]. In contrast, 6-BA directly provides adenine-type cytokinin activity that supports shoot organogenesis and subsequent proliferation [38]. The synergistic effect of combining TDZ and 6-BA has been recognized as a critical factor in optimizing tissue culture systems for diverse woody species, including mulberry (*Morus* spp.) [39] and others [28,40]. In mulberry, TDZ was shown to rearrange adenine-type cytokinin, downregulate endogenous cytokinin signaling, and enhance auxin signaling by upregulating related genes without altering auxin biosynthesis [39]. It should be noted that a negative control (PGR-free medium) was not included in the present experiment for shoot regeneration from hypocotyls, which limits the ability to assess baseline shoot regeneration. Future studies should include such controls to validate the necessity of exogenous PGRs.

Light plays a pivotal role in regulating plant growth and morphogenic processes, including in vitro regeneration. The explants of plant species complete their regeneration process by responding to different photoreceptors under various light spectra [41,42]. Photoreceptors transmit light signals to downstream regulatory factors, such as PHYTOCHROME-INTERACTING FACTORS (PIFs), ELONGATED HYOCOTYL 5 (HY5), and CONSTITUTIVE PHOTOMORPHOGENIC 1 (COP1) [43]. These conserved light-responsive signaling factors then regulate downstream genes and proteins involved in plant regeneration, including the cytokinin-responsive factor ARABIDOPSIS RESPONSE REGULATOR 12 (ARR12) and WUSCHEL (WUS) of Arabidopsis [44]; auxin synthesis genes, such as AMIDASE 1 (AMI1); and jasmonic acid (JA)-responsive genes, such as DE-ETIOLATED-2 (DET2) [45,46]. Studies have shown that blue [47], red [48], or a combination of red and blue light [49] can significantly promote shoot regeneration efficacy for different plant species. Our results showed that white LED light was most effective for adventitious shoot regeneration in *H. chrysanthus*, while green light completely suppressed organogenesis. This finding is consistent with previous reports in other woody species. For instance, white or yellow light promoted adventitious shoot regeneration in *Populus alba* × *P. berolinensis*, whereas green light inhibited this process [50].

Hyperhydricity represents one of the most pervasive physiological disorders in plant tissue culture [51,52]. Hyperhydricity is also a common challenge during in vitro propagation of *Handroanthus* species [53,54]. During the in vitro regeneration of *H. chrysanthus*, approximately 10–15% of the regenerated shoots exhibited varying degrees of hyperhydricity, as shown in Figure 2b. Anatomical studies on *H. impetiginosus* revealed that hyperhydric shoots exhibit severe structural abnormalities at the proliferation stage, including disorganized cortex, epidermal discontinuity, collapsed cells, and stomatal malformation—defects that directly compromise survival during acclimatization [53]. High concentrations of cytokinins—commonly used to maximize shoot proliferation—are well-established inducers of hyperhydricity, whereas reducing cytokinin levels in the medium can effectively alleviate this disorder [55]. The moderate multiplication coefficient of 3.27 achieved in our study represents a pragmatic compromise. In a recent study, Grira [54] reduced the hyperhydricity of *H. guayacan* by increasing gelling agent (agar or Gelrite) concentration, optimizing sucrose and cytokinin levels, and supplementing with calcium, silicon, and anti-ethylene compounds (AgNO_3_). Meanwhile, alternative cytokinin types—particularly meta-topolin (mT) and its riboside (mTR)—have also shown promise in reducing hyperhydricity while maintaining efficient shoot multiplication in *H. guayacan*, *T. rosea*, *Tectona grandis*, and wild olive. Collectively, these strategies offer a promising approach for refining regeneration systems in *Handroanthus* species.

## 4. Materials and Methods

### 4.1. Plant Material and Explant Preparation

Winged seeds of *H. chrysanthus* were collected from mature trees located at South China Agricultural University, Guangzhou, China. Seeds were initially cut off the wings and washed with a mild detergent solution for 15 min, followed by thorough rinsing under running tap water for 30 min. Surface sterilization was conducted by immersing the seeds in 75% (*v*/*v*) ethanol for 50 s, followed by three rinses with sterile distilled water. Subsequently, seeds were treated with 0.1% (*w*/*v*) HgCl_2_ for varying durations (6, 8, 10, 12, 14, 16 min) and rinsed five times with sterile distilled water. Sterilized seeds were placed on hormone-free Murashige and Skoog (MS) basal medium for germination. After 20 days, hypocotyls and cotyledons were excised from aseptic seedlings and used as explants for regeneration experiments.

### 4.2. Culture Media and Conditions

The basal media used in this study included MS [25], 1/2 MS, and DCR [33] (Douglas-fir Cotyledon Revised Medium). MS medium (HB8469-5) and 1/2 MS medium (HB8469-12) were supplied by Qingdao Hopebio Bio-Technology Co., Ltd. (Qingdao, China), whereas DCR medium (PM1631) was obtained from Coolaber Science and Technology Co., Ltd. (Beijing, China). All media were supplemented with 30 g·L^−1^ (for micropropagation) or 20 g·L^−1^ (for regeneration) sucrose and solidified with 6.0 g·L^−1^ agar (for micropropagation) or 2.4 g·L^−1^ phytagel (for regeneration). The pH was adjusted to 5.8 prior to autoclaving at 121 °C for 20 min. Cultures were maintained in a growth room at 25 ± 2 °C under a 16 h photoperiod provided by white LED light (50 µmol·m^−2^·s^−1^ photosynthetic photon flux density). In the light quality experiment, the photosynthetic photon flux density (PPFD) was maintained constant at 50 µmol·m^−2^·s^−1^ under all light spectra tested (white LED, red, green, and blue). LED tubes were purchased from iGrowLite (Guangzhou Zhiui Guangtian Agricultural Technology Co., Ltd., Guangzhou, China), model IGL-T5-10W-S, with the following peak wavelengths: white (400–700 nm), red (660 nm), green (520 nm), and blue (450 nm).

Preliminary experiments were conducted to select the optimal basal medium for each explant type. For hypocotyl explants, MS, 1/2 MS, WPM, and DCR were compared. Based on the results of previous studies on the related species *H. impetiginosa* ‘Zi Xiuqiu’ in our laboratory [30], MS medium was found to be the most suitable for hypocotyl regeneration and was therefore used for all formal hypocotyl experiments. For cotyledon explants, preliminary tests showed that explants failed to grow on MS medium, while shoot formation was observed only on DCR medium. Consequently, DCR medium was used exclusively for cotyledon regeneration experiments.

### 4.3. Adventitious Shoot Regeneration from Hypocotyls

Hypocotyl segments (approximately 0.8–1.0 cm in length) were excised and placed horizontally on MS basal medium supplemented with different concentrations of TDZ (thidiazuron), 6-BA (6-benzylaminopurine), NAA (1-naphthaleneacetic acid), and IBA (indole-3-butyric acid). For the 6-BA + NAA growth regulator combination, the concentration of 6-BA was 3, 5, or 7 mg·L^−1^, while NAA was tested at 0, 0.05, 0.1, 0.2, 0.4, or 0.5 mg·L^−1^. These treatments were designated as SR1 to SR13, with the specific concentrations detailed in Table 2. For the TDZ + 6-BA + IBA combination, a two-factor factorial design was used. TDZ concentrations tested were 0.2, 0.5, 1.0, 2.0, and 3.0 mg·L^−1^; 6-BA concentrations were 0, 0.5, 1.0, 1.5, 3.0, and 5.0 mg·L^−1^; IBA was tested at 0.3 and 0.5 mg·L^−1^. Selected combinations (SI1–SI13) representing the full range of responses are presented in Table 3. Each treatment consisted of 30 explants. Data on callus formation, shoot regeneration frequency, and the number of shoots per explant were recorded after 20, 45, and 60 days of culture. BAP (DH038-2), IBA (II172) and NAA (IN211) were purchased from Beijing Dingguo Chang-sheng Biotechnology Co., Ltd. (Beijing, China), and TDZ (P6186) was purchased from Sigma-Aldrich (St. Louis, MO, USA).

### 4.4. Adventitious Shoot Regeneration from Cotyledons

Cotyledons from 20-day-old seedlings were placed abaxially on MS or DCR medium containing 6-BA (3, 5, or 7 mg·L^−1^) and NAA (0, 0.05, or 0.5 mg·L^−1^). Each treatment comprised 15 explants with three replicates. After 60 days, data on callus formation, shoot regeneration rate, and induced shoot number per explant were recorded and analyzed.

### 4.5. Shoot Proliferation

Regenerated shoots (1.5–2.0 cm in length) were transferred to MS medium supplemented with 6-BA (2, 3, or 4 mg·L^−1^) and gibberellic acid (GA_3_; 0.1, 0.3, or 0.5 mg·L^−1^) for proliferation. Each treatment involved 15 explants and was repeated three times. After 45 days of subculture, the proliferation coefficient was calculated as the number of newly formed adventitious shoots (≥0.5 cm in height) per explant, excluding any shoots that were already present on the original explant.

### 4.6. Rooting and Acclimatization

In vitro-derived shoots (2–3 cm long) were transferred to half-strength MS medium supplemented with different concentrations of IBA (0, 0.01, 0.05, and 0.5 mg·L^−1^) and NAA (3, 5, and 10 mg·L^−1^), with or without 0.2 g·L^−1^ of activated charcoal (A.C.). Each treatment involved 15 explants and was repeated three times. Rooting percentage, root number, and root length were recorded after 30 days. Well-rooted plantlets were acclimatized in a greenhouse using a sterilized substrate mixture of peat, vermiculite, and perlite (3:1:1 *v*/*v*/*v*). The survival rate was assessed after one month.

### 4.7. Statistical Analysis

All experiments were arranged in a completely randomized design with three replicates per treatment. Before one-way or two-way ANOVA, data were tested for normality (Shapiro–Wilk test, *p* > 0.05) and homogeneity of variances (Levene’s test, *p* > 0.05). Percentage data (e.g., shoot regeneration rate, rooting percentage) were arcsine square-root transformed when necessary to satisfy ANOVA assumptions, whereas untransformed means are presented in the tables. Data were subjected to analysis of variance (ANOVA) using SPSS 26.0 software. Mean separations were performed using Duncan’s multiple-range test at a significance level of *p* < 0.05. All explants were randomly assigned to each treatment. Three replicates refer to three independent biological replicates.

## 5. Conclusions

In this study, we established a reliable and efficient direct adventitious shoot regeneration system for *H. chrysanthus* using hypocotyl explants. The key findings can be summarized as follows: (1) Explant type is critical—hypocotyls showed significantly higher regenerative capacity than cotyledons. (2) TDZ is a key regulator—its combination with 6-BA and IBA synergistically enhanced shoot induction. (3) Light quality matters—white LED light was optimal for shoot regeneration. (4) Ex vitro acclimatization is achievable—over 95% of regenerated plantlets survived in the greenhouse.

This regeneration system provides a technical platform for several future applications. First, it enables the mass propagation of *H. chrysanthus* to meet horticultural demand and support ex situ conservation efforts for this vulnerable species. Second, it serves as a foundation for genetic improvement—the efficient adventitious shoot induction is a prerequisite for Agrobacterium-mediated transformation and CRISPR/Cas9 gene editing, which could be used to improve undesirable traits such as a short flowering period and cold sensitivity. Third, the protocols developed here offer a technical reference for other endangered species within the Tabebuia alliance (e.g., *H. heptaphyllus*, *H. impetiginosus*), facilitating their regeneration and conservation.

Collectively, this work transforms a species-specific regeneration protocol into a broader strategy for combining biotechnology with conservation and breeding of non-model woody plants.

## Figures and Tables

**Table 1:** Effect of 0.1% HgCl_2_ sterilization duration on aseptic seedling establishment of *Handroanthus chrysanthus*.

Treatment 0.1% HgCl_2_ (min)	Contamination Rate (%)	Germination Rate (%)
6	21.75 ± 3.31 a	72.20 ± 4.20 bc
8	15.21 ± 2.45 b	76.30 ± 1.69 ab
10	10.20 ± 3.22 c	86.32 ± 1.80 a
12	8.20 ± 3.37 c	84.32 ± 3.20 a
14	8.07 ± 1.78 c	78.20 ± 2.10 ab
16	3.33 ± 0.50 d	65.70 ± 3.51 c

Note: Different lowercase letters in the same column represent significant differences at the 0.05 level according to Duncan’s multiple-range test.

**Table 2:** Effect of 6-BA and NAA combination on the induction of callus and shoot regeneration from the hypocotyls of *Handroanthus chrysanthus* after 45 days.

Treatments	6-BA (mg⋅L^−1^)	NAA (mg⋅L^−1^)	Callus Formation (%)	Shoot Regeneration (%)	Shoots per Explant
SR1	3	0	59.31 ± 8.07 f	8.10 ± 4.23 abc	0.67 ± 0.33 ab
SR2	3	0.05	98.33 ± 2.89 ab	14.26 ± 1.65 abc	1.11 ± 0.19 ab
SR3	3	0.5	86.75 ± 4.86 cd	7.90 ± 0.21 abc	1.33 ± 0.58 a
SR4	5	0	66.77 ± 3.18 e	6.36 ± 3.19 bc	0.67 ± 0.58 ab
SR5	5	0.05	100.00 ± 0.00 a	20.50 ± 3.21 a	1.00 ± 0.00 ab
SR6	5	0.1	100.00 ± 0.00 a	19.98 ± 5.54 ab	1.11 ± 0.19 ab
SR7	5	0.2	100.00 ± 0.00 a	16.38 ± 1.13 ab	1.00 ± 0.00 ab
SR8	5	0.4	100.00 ± 0.00 a	7.87 ± 3.96 abc	0.67 ± 0.58 ab
SR9	5	0.5	79.87 ± 2.02 d	3.33 ± 3.33 c	0.33 ± 0.58 b
SR10	7	0.05	100.00 ± 0.00 a	16.03 ± 3.31 ab	1.00 ± 0.00 ab
SR11	7	0.1	95.83 ± 7.22 ab	10.83 ± 5.83 abc	0.67 ± 0.58 ab
SR12	7	0.2	91.07 ± 7.79 bc	8.33 ± 4.17 abc	0.67 ± 0.58 ab
SR13	7	0.4	100.00 ± 0.00 a	10.83 ± 5.83 abc	0.67 ± 0.58 ab

Note: Different lowercase letters in the same column represent significant differences at the 0.05 level according to Duncan’s multiple-range test.

**Table 3:** Effects of 6-BA, TDZ, and IBA combinations on the regeneration of adventitious shoots from the hypocotyls of *Handroanthus chrysanthus*.

Treatments	6-BA (mg⋅L^−1^)	TDZ (mg⋅L^−1^)	IBA (mg⋅L^−1^)	Callus Formation (%)	Shoot Regeneration (%)	Shoots per Explant
SI1	0	0.2	0.5	100.00 ± 0.00 a	15.08 ± 0.79 bcd	0.67 ± 0.578 ab
SI2	0.5	0.2	0.5	100.00 ± 0.00 a	15.74 ± 7.91 bcd	1.22 ± 0.19 a
SI3	1	0.2	0.5	100.00 ± 0.00 a	29.37 ± 7.57 b	1.00 ± 1.00 ab
SI4	1.5	0.5	0.3	79.33 ± 5.10 c	8.46 ± 4.33 d	0.67 ± 0.58 ab
SI5	1.5	0.5	0.5	100.00 ± 0.00 a	17.50 ± 2.50 bcd	0.67 ± 0.58 ab
SI6	1.5	1	0.3	100.00 ± 0.00 a	3.33 ± 3.33 d	0.33 ± 0.58 b
SI7	1.5	1	0.5	100.00 ± 0.00 a	14.44 ± 5.30 bcd	1.00 ± 1.00 ab
SI8	1.5	2	0.3	100.00 ± 0.00 a	4.76 ± 4.76 d	0.33 ± 0.58 b
SI9	1.5	2	0.5	92.80 ± 6.46 b	12.12 ± 6.06 bcd	1.00 ± 0.00 ab
SI10	1.5	3	0.3	82.22 ± 5.88 c	10.74 ± 6.43 cd	0.33 ± 0.58 b
SI11	1.5	3	0.5	100.00 ± 0.00 a	6.06 ± 3.03 d	0.67 ± 0.58 ab
SI12	3	0.2	0.5	100.00 ± 0.00 a	30.00 ± 0.00 b	1.28 ± 0.25 a
SI13	5	0.2	0.5	100.00 ± 0.00 a	51.79 ± 10.91 a	2.22 ± 0.47 a

Note: Different lowercase letters in the same column represent significant differences at the 0.05 level.

**Table 4:** Effect of light spectrum on the regeneration of adventitious shoots from hypocotyl explants of *Handroanthus chrysanthus*.

Light Spectrum	Callus Induction Rate (%)	Shoot Regeneration Rate (%)	Shoots per Explant
White LED	100.00 ± 0	51.79 ± 9.94 a	2.20 ± 0.20 a
Red	100.00 ± 0	6.67 ± 5.78 b	0.67 ± 0.58 b
Green	100.00 ± 0	0 ± 0 c	0 ± 0 c
Blue	100.00 ± 0	10.00 ± 0 b	1.00 ± 0 b

Note: Different lowercase letters in the same column represent significant differences at the 0.05 level according to Duncan’s multiple-range test.

**Table 5:** Adventitious bud induction of *Handroanthus chrysanthus* cotyledons in different culture media.

Treatments	6-BA (mg⋅L^−1^)	NAA (mg⋅L^−1^)	Callus Formation (%)	Shoot Regeneration (%)	Shoots per Explant
DC1	3	0.05	100.00 ± 0.00 a	0.00 ± 0.00 b	0.00 ± 0.00 c
DC2	3	0.3	100.00 ± 0.00 a	10.87 ± 3.07 a	1.33 ± 0.58 a
DC3	3	0.5	100.00 ± 0.00 a	0.00 ± 0.00 b	0.00 ± 0.00 c
DC4	5	0.05	91.11 ± 1.92 ab	25.00 ± 5.88 a	1.08 ± 0.95 ab
DC5	5	0.3	86.11 ± 7.34 b	0.00 ± 0.00 b	0.00 ± 0.00 c
DC6	5	0.5	100.00 ± 0.00 a	0.00 ± 0.00 b	0.00 ± 0.00 c
DC7	7	0.05	83.21 ± 7.60 b	5.34 ± 4.64 ab	0.67 ± 0.58 b
DC8	7	0.3	100.00 ± 0.00 a	3.03 ± 5.25 ab	0.33 ± 0.58 bc
DC9	7	0.5	89.68 ± 5.06 ab	2.78 ± 4.81 ab	0.33 ± 0.58 bc

Note: Different lowercase letters in the same column represent significant differences at the 0.05 level.

**Table 6:** The effects of 6-BA and GA_3_ combinations on shoot proliferation of *Handroanthus chrysanthus*.

Treatments	6-BA (mg⋅L^−1^)	GA3 (mg⋅L^−1^)	Adventitious Bud Induction Rate (%)	Proliferation Co-Efficiency
SP1	2	0.1	85.35 ± 2.81 ab	2.16 ± 0.17 c
SP2	3	0.1	66.67 ± 4.81 cd	2.50 ± 0.08 bc
SP3	4	0.1	80.56 ± 2.78 abc	2.56 ± 0.19 bc
SP4	2	0.3	70.45 ± 6.48 bc	2.35 ± 0.44 bc
SP5	3	0.3	52.27 ± 11.67 de	2.71 ± 0.02 b
SP6	4	0.3	91.67 ± 0.00 a	3.27 ± 0.05 a
SP7	2	0.5	77.88 ± 6.56 abc	2.20 ± 0.21 c
SP8	3	0.5	51.26 ± 3.77 e	2.68 ± 0.14 b
SP9	4	0.5	41.67 ± 4.81 e	2.56 ± 0.13 b

Note: Different lowercase letters in the same column represent significant differences at the 0.05 level. The proliferation coefficient refers to the total number of adventitious shoots (≥0.5 cm in height) regenerated per explant, excluding shoots present on the original explant.

**Table 7:** Effect of NAA and IBA on root induction and growth of *Handroanthus chrysanthus*.

Treatments	NAA (mg⋅L^−1^)	IBA (mg⋅L^−1^)	Rooting Percentage (%)	Root Length (cm)	Root Number/Plant
R1	0	3	27.78 ± 5.56 def	1.83 ± 1.04 de	1.33 ± 1.00 bc
R2	0	5	33.33 ± 9.62 cde	3.12 ± 0.33 bcd	5.44 ± 3.15 ab
R3	0	10	33.33 ± 0.00 cde	4.56 ± 2.87 ab	2.75 ± 2.54 abc
R4	0	15	8.33 ± 8.33 f	0.63 ± 1.10 e	0.67 ± 1.16 c
R5	0.01	3	66.67 ± 9.62 ab	3.27 ± 1.77 bcd	3.66 ± 1.94 abc
R6	0.01	5	33.33 ± 8.33 cde	2.41 ± 1.97 cd	3.66 ± 2.08 abc
R7	0.01	10	38.89 ± 5.56 cd	2.62 ± 2.08 cd	3.38 ± 3.39 abc
R8	0.01	15	55.56 ± 5.56 bc	1.18 ± 0.25 de	3.41 ± 1.66 abc
R9	0.05	3	33.33 ± 6.67 cde	1.70 ± 1.44 de	2.67 ± 2.02 bc
R10	0.05	5	40.00 ± 0.00 cd	2.52 ± 1.43 cd	1.50 ± 1.07 bc
R11	0.05	10	66.67 ± 6.67 ab	5.27 ± 1.65 a	3.97 ± 0.55 abc
R12	0.05	15	46.67 ± 6.67 bcd	2.43 ± 1.21 cd	7.28 ± 5.09 a
R13	0.5	3	50.00 ± 9.62 bcd	3.27 ± 1.63 bcd	1.75 ± 1.75 bc
R14	0.5	5	80.00 ± 11.54 a	4.63 ± 1.31 ab	1.93 ± 2.63 bc
R15	0.5	10	55.56 ± 9.55 bc	3.92 ± 0.78 abc	2.28 ± 0.25 bc
R16	0.5	15	66.67 ± 9.62 ab	4.62 ± 1.09 ab	1.98 ± 1.37 bc

Note: Different lowercase letters in the same column represent significant differences at the 0.05 level.

## Data Availability

The original contributions presented in the study are included in the article; further inquiries can be directed to the corresponding author.
